# Targeting Epithelial–Mesenchymal Transition (EMT) to Overcome Drug Resistance in Cancer

**DOI:** 10.3390/molecules21070965

**Published:** 2016-07-22

**Authors:** Bowen Du, Joong Sup Shim

**Affiliations:** Faculty of Health Sciences, University of Macau, Avenida da Universidade, Taipa, Macau SAR 999078, China; dbw@mail.ustc.edu.cn

**Keywords:** epithelial–mesenchymal transition, drug resistance, chemotherapy, cancer stem cells

## Abstract

Epithelial–mesenchymal transition (EMT) is known to play an important role in cancer progression, metastasis and drug resistance. Although there are controversies surrounding the causal relationship between EMT and cancer metastasis, the role of EMT in cancer drug resistance has been increasingly recognized. Numerous EMT-related signaling pathways are involved in drug resistance in cancer cells. Cells undergoing EMT show a feature similar to cancer stem cells (CSCs), such as an increase in drug efflux pumps and anti-apoptotic effects. Therefore, targeting EMT has been considered a novel opportunity to overcome cancer drug resistance. This review describes the mechanism by which EMT contributes to drug resistance in cancer cells and summarizes new advances in research in EMT-associated drug resistance.

## 1. Introduction

Cancer is one of the leading causes of human death. Despite significant advances in cancer research throughout the decades, treatment of cancer is still facing serious challenges. Chemotherapy is one of the well-established types of cancer treatment and has long been used as monotherapy or in combination with surgery or radiotherapy to treat cancer patients. However, chemotherapy drugs, both classical cytotoxic drugs and molecular targeted drugs, have been challenged by drug resistance, a major cause of cancer treatment failure and cancer-related mortality. In the last decade, tremendous effort has been paid to develop targeted cancer therapies. A number of monoclonal antibody drugs and small molecules, especially kinase inhibitors, have been developed and entered clinic in the hope of improving anticancer efficacy. While many of the targeted therapy drugs showed promising early clinical outcomes with improved overall survival, a large number of the patients receiving targeted therapy developed drug resistance after long-term drug administration [1]. Currently, more than 100 targeted cancer therapy drugs have been approved for cancer patients and much more are in clinical investigations. Therefore, cancer drug resistance will be a key factor to determine the success of the upcoming targeted therapy drugs. Drug resistance (or chemoresistance) can be divided into two groups: intrinsic (or de novo) drug resistance and acquired drug resistance [2,3]. Intrinsic drug resistance refers that the resistance factors have existed in the bulk of tumor cells before the drug treatment, whereas acquired drug resistance comes from that the resistance factors are developed during the drug treatment. Drug resistance arises from a broad range of mechanisms, such as drug efflux, drug metabolism, drug target mutations, etc. Recently, epithelial–mesenchymal transition (EMT) has received increasing attention for its role in cancer drug resistance. This review will focus on the link between EMT and drug resistance.

## 2. EMT-Related Signaling Pathways

**Figure 1:**
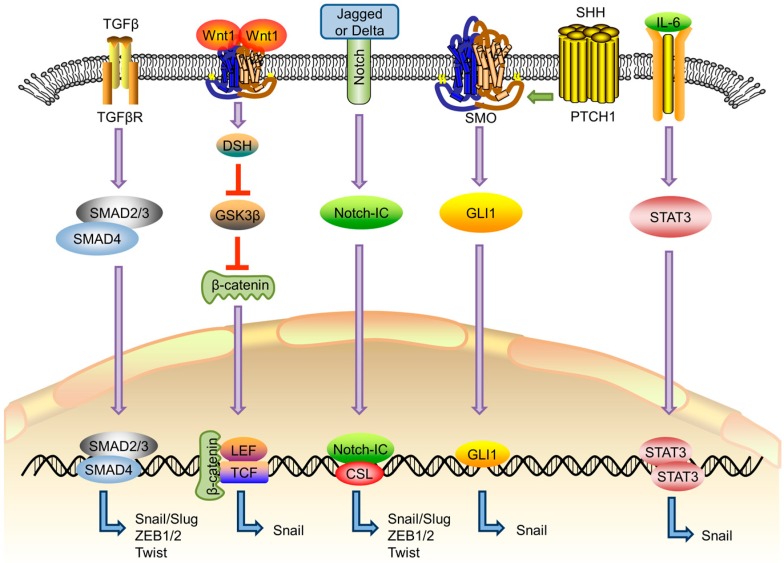
Diverse signaling pathways associated with epithelial–mesenchymal transition (EMT). Transforming growth factor beta (TGFβ) signals activate SMAD2 and SMAD3 that complex with SMAD4. The trimeric SMAD complex enters the nucleus and leads to the transcription of EMT transcription factors (EMT-TFs) [9]. Activation of Wnt signaling inhibits the destruction complex containing glycogen synthase kinase 3 beta (GSK-3β) through Disheveled (DSH), facilitating β-catenin to enter the nucleus and activate the Snail transcription [10]. Notch receptors can be activated by binding to Delta and Jagged ligands. After activation, Notch intracellular domain (Notch-IC) is released through a cascade of proteolytic cleavages and activates CSL transcription factor to express EMT-TFs [11]. In Sonic Hedgehog (SHH) signaling, ligand binding to Patched 1 (PTCH1) receptors activates Smoothened (SMO) and Glioma (GLI) family transcription factors that induce Snail expression [12]. Interleukin-6 (IL-6) can induce Snail expression by activating STAT3 [13].

EMT is the process that epithelial cells lose the apical-basal polarity and cell–cell adhesion, and transit to invasive mesenchymal cells. Mesenchymal–epithelial transition (MET), on the other hand, is the reverse process of EMT that involves the transition of cells from motile, multipolar mesenchymal types to polarized epithelial types. EMT are involved in numerous biological and pathological processes, including embryonic development, wound healing, cancer cell metastasis and drug resistance [4,5,6]. EMT refers to diverse changes in cells at a molecular level. Cells undergoing EMT display decreased expression level of epithelial genes (such as E-cadherin, ZO-1 and occludin) and increased expression level of mesenchymal genes (such as N-cadherin, vimentin and fibronectin) [7]. In most cases, loss of E-cadherin is a hallmark of EMT. The changes in gene expression during EMT lead to numerous phenotypic changes, such as cell morphological changes, loss of adhesion and gain of stem cell-like features [7]. Several key signaling pathways, including transforming growth factor beta (TGFβ), Wnt, Notch and Hedgehog, are known to be involved in EMT [8]. Signaling pathways that are related to EMT and their transcription targets that play a critical role in EMT are summarized in Figure 1. TGFβ pathway can be activated by binding TGFβ superfamily of ligands, including TGFβs, bone morphogenetic proteins (BMPs) and Nodal, to their cognate TGFβ receptors. Upon stimulation of TGFβ receptors, TGFβ signaling is divided into SMAD-dependent and SMAD-independent pathways. In the SMAD-dependent pathway, expression of the mesenchymal genes, such as vimentin, can be induced by SMAD complex upon activation of TGFβ receptor [9].

In the SMAD-independent pathway, TGFβ signal can induce EMT through GTPases, PI3K and MAPK pathways [14]. Other signaling pathways, including Notch [11], Wnt [10], Hedgehog [12], AKT-mTOR [15], MAPK/ERK [16], NF-κB [17] pathways, are also involved in EMT. These signaling pathways ultimately lead to an activation of EMT transcription factors (EMT-TFs). Several transcription factors have been identified as master regulators of EMT, including SNAIL factors (SNAI1, also known as Snail and SNAI2, also known as Slug), bHLH factors (E12 and E47, TWIST1 and TWIST2) and ZEB factors (ZEB1 and ZEB2) [7]. There are also some newly discovered EMT-TFs, such as SOX and FOX transcription factors. These EMT-TFs bind to specific DNA sequences, such as E-box to regulate EMT-target genes [7]. E-cadherin is a well-known transcription target for repression by Snail and many other EMT-TFs [18,19,20]. In addition to E-cadherin, a number of key proteins that are involved in epithelial cell–cell junction formation and cytoskeletal and polarity complex proteins are major transcription targets of EMT-TFs [7]. Epithelial cell–cell junction proteins, including occludin, claudins and desmoplakin as well as E-cadherin are targets for transcription repression, and cytoskeletal and polarity complex proteins, such as fibronectin, vitronectin, vimentin, N-cadherin and matrix metalloproteinases (MMPs) are targets for transcription activation [21,22,23].

## 3. EMT and Cancer Drug Resistance

The link between EMT and cancer cell drug resistance has been suggested in the early 1990s. Sommers et al. found that two adriamycin-resistant MCF-7 cell lines and a vinblastine-resistant ZR-75-B cell line underwent EMT [24]. Adriamycin-resistant MCF-7 cells showed a significant increase in vimentin expression and have reduced formation of desmosomes and tight junctions, a typical phenotype in EMT. Not all of the drug-resistant MCF-7 cells exhibited EMT phenotypes, suggesting that among the heterogenic cancer cell population, EMT cells have selective growth advantage in the presence of drugs. It has been increasingly recognized that cancer drug resistance is frequently accompanied with EMT in diverse cancer, including pancreatic cancer [25], bladder cancer [26] and breast cancer [27]. Very recently, two research groups demonstrated a causal relationship between EMT and cancer drug resistance using genetically-engineered mice models [28,29]. Fischer et al. [28] established an EMT lineage-tracing system to monitor reversible and transient EMT process in mice. They generated Fsp1 (fibroblast specific protein 1) promoter-driven Cre recombinase that can specifically activate GFP expression upon lox cleavage. This system allowed to trace cancer cells that underwent EMT, even after metastasized and turned to epithelial phenotype (MET) again. Upon treatment with cancer chemotherapy drug cyclophosphamide, primary tumor growth was reduced by 60% in this mouse model. However, GFP-positive EMT cells in the primary tumor showed resistant to apoptosis induction and no significant reduction in cell number under chemotherapy treatment compared to epithelial type cancer cells. Strikingly, in the control group of mice, most of the lung metastasized cancer cells had not undergone EMT, while chemotherapy treated mice had significantly larger number of EMT cancer cells in lung metastatic region. These data suggested that EMT plays an important role in cancer drug resistance and contributes to metastasis after chemotherapy treatment.

Signaling pathways that promote EMT phenotype were actually known to contribute to drug resistance. For instance, TGFβ, a well-studied EMT-related cytokine, was reported to be related with drug resistance in 1990s. Teicher et al. found that TGFβ-neutralizing antibodies restored drug sensitivity in the alkylating agent-resistant tumors [30]. Further studies demonstrated that TGFβ induced EMT and this in turn led to the drug resistance. Doxorubicin has been observed to induce expression of circulating TGFβ in animal models [31]. Colon cancer cells treated with doxorubicin underwent EMT, and inhibition of TGFβ/Smad4 signaling pathway by the downregulation of Smad4 reversed doxorubicin-induced EMT [32]. Wnt and Hedgehog pathways are also known to contribute to drug resistance. Overexpression of Wnt3 activated Wnt/β-catenin signaling pathway and promoted EMT, which led to a trastuzumab-resistant phenotype in human epidermal growth factor receptor 2 (HER2)-overexpressing breast cancer cells [33]. Activation of Hedgehog pathway could mediate EGF receptor tyrosine kinase inhibitor (EGFR-TKI) resistance by inducing EMT in lung cancer cells [34]. EMT-TFs are also known to promote cancer drug resistance. Overexpression of Twist induced EMT and promoted the resistance of colorectal cancer cells to oxaliplatin treatment by increasing multidrug resistance protein 1 (MDR1) [35]. FOX transcription factor superfamily members seem to have different effects on drug resistance. FOXC2 and FOXM1 promoted drug resistance [36,37], whereas FOXF2 suppressed FOXC2-mediated EMT [38]. Additionally, other EMT-TFs, such as Snail, Slug and ZEB, were also reported to be related to drug resistance [39,40].

## 4. Mechanism of EMT-Induced Drug Resistance

The link between EMT and drug resistance has been reported for a long time, but the mechanism is still elusive. Benefiting from fast-moving cancer stem cell (CSC) research, scientists have a new insight into the mechanism of drug resistance in cells undergoing EMT. CSCs are a small sub-population of cells in the bulk tumor cells, which are responsible for tumorigenesis [41]. There are remarkable similarities in signaling pathways between those activated during EMT and those driving CSC, such as Wnt, Hedgehog and Notch signaling pathways. These pathways are critical for CSC self-renewal and maintenance [42]. Empirical evidence suggested that cells undergone EMT have a stem cell-like property, thus sharing key signaling pathways and drug resistance phenotypes with CSCs [43,44]. However, EMT is unlikely a prerequisite for cancer cell stemness as later findings demonstrated the presence of heterogenic CSC populations, including mesenchymal-like CSCs (EMT CSCs) and epithelial-like CSCs (non-EMT CSCs) [45,46,47]. One of the main mechanisms of drug resistance in CSCs is excessive drug efflux by multiple cell membrane transporter proteins, especially, the ATP-binding cassette (ABC) transporter family of proteins. At least three members of ABC transporter family, including MDR1 (also known as P-glycoprotein and ABCB1), MDR-associated protein 1 (MRP1; also known as ABCC1) and breast cancer resistance protein (BCRP; also known as ABCG2) are known to be involved in drug resistance [48]. These transporters are membrane proteins with broad substrate specificity [49,50,51], thus responsible for diverse drug resistance. Cells undergoing EMT overexpressed ABC transporters and showed drug resistance phenotype similar to CSCs. Saxena et al., demonstrated that promoters of ABC transporters contain several binding sites for EMT-TFs [52]. Overexpression of EMT-TFs such as Twist, Snail and FOXC2 increased the promoter activity and the expression of ABC transporters in breast cancer cells. These cells showed 10-fold higher resistance to doxorubicin treatment compared with the control, non-transfected cells [52].

Another important mechanism underlying EMT-driven drug resistance is the gain of cellular resistance to drug-induced apoptosis. EGFR-TKIs, such as erlotinib and gefitinib, are commonly used for the treatment of non-small cell lung cancer (NSCLC). EGFR-TKIs can bind to the ATP-binding site of EGFRs, thus inhibiting its activity and inducing cell apoptosis. Several groups have reported that EMT could induce cancer cell resistance to EGFR-TKI [53,54], whereas restoring E-cadherin expression increased cancer cell sensitivity to EGFR-TKIs [55]. Growing evidence suggests that EMT-TFs can inhibit EGFR-TKI-induced apoptosis. Slug is believed to confer gefitinib resistance by suppressing Bim expression and enhancing caspase-9 activity in NSCLC [56]. Notch-1 overexpression is associated with EMT in gefitinib-acquired resistance and protects EGFR-mutant cells from gefitinib-induced apoptosis [57]. However, some contradictory findings have been reported recently. Using human NSCLC lines and a transgenic lung cancer mouse model harboring mutant EGFR, Soucheray et al., showed that chronic exposure of NSCLC to EGFR-TKIs activated TGFβ/SMAD signaling and promoted EMT phenotype [58]. However, combined inhibition of EGFR and TGFβ receptor prevented EMT but could not prevent drug resistance. This study suggested that the presence of intratumoral heterogeneity led to divergent resistance mechanisms and that inhibition of EMT alone might not be sufficient to prevent the drug resistant phenotype of NSCLC to EGFR-TKIs. This notion was further supported by the latest report published by Yoshida et al. This group showed that ZEB1 mediated EMT-related acquired resistance of NSCLC to the EGFR-TKIs. However, none of EMT inhibitors could completely re-sensitize NSCLC cells to the EGFR-TKIs, suggesting that EMT inhibition may not be sufficient to restore drug resistant phenotype to the EGFR-TKIs [59].

Tumor microenvironment is also a factor mediating EMT-driven drug resistance. Cancer-associated fibroblasts (CAF), a component of tumor stroma, play an important role in supporting the proliferative and invasive behavior of cancer cells through cell–cell interaction or extracellular signaling molecules [60]. These fibroblasts facilitate tumor cells to undergo EMT through secretion of cytokines, such as IL-6 and TCF21 [61,62]. Hypoxia is another important tumor microenvironment that promotes cancer cells to undergo EMT and acquire drug resistance. Activation of HIF-1α under hypoxic condition promoted hepatocellular carcinoma (HCC) to EMT and induced drug resistance by increasing the expression of MDR1 [63]. Knock-down of HIF-1α reversed EMT phenotype and abolished drug resistant phenotype of HCC under hypoxia, further supporting the role of hypoxia/HIF-1α in EMT-driven drug resistance.

## 5. Overcoming Drug Resistance by Targeting EMT

Earlier in the 1990s, a calcium channel blocker verapamil was tested in clinical trials as a chemosensitizer to reverse drug resistance, as this drug was known to inhibit MDR1 [64]. However, verapamil showed no beneficial effect on VAD (vincristine, doxorubicin and dexamethasone combination) chemotherapy regimen for the treatment of drug-resistant myeloma patients, mainly due to the dose-limiting toxicity [65]. Nevertheless, extensive efforts have been paid to overcome drug resistance by directly targeting the ABC transporters [66,67]. Since it has been increasingly evidenced that EMT plays an important role in drug resistance, scientists started looking into drugs targeting EMT to overcome drug resistance. Gupta et al. generated EMT cells by introducing E-cadherin shRNA and used this cell line to identify CSC-selective small molecule inhibitors. Through a high throughput screening, they identified an antibiotic salinomycin that selectively killed breast CSCs [68]. Further study demonstrated that salinomycin inhibited EMT that was induced by doxorubicin treatment and enhanced doxorubicin sensitivity in HCC cells [69]. Salinomycin also reduced doxorubicin resistance by diminishing drug efflux pump expression and activity in breast cancer cells [70]. In addition to salinomycin, a number of small molecule inhibitors of EMT have been identified and tested in vitro and in vivo models of cancer drug resistance. Curcumin, an active ingredient in curry was shown to sensitize colorectal cancer cells that were resistant to 5-fluorouracil through miRNA-mediated suppression of EMT [71]. A histone deacetylase (HDAC) inhibitor mocetinostat inhibited expression of an EMT-TF, ZEB1 by restoring miR-203, reversed the EMT phenotype in drug resistant pancreatic cancer cells and sensitized the cells to a chemotherapy drug docetaxel [72]. Namba et al., reported that Akt/GSK3β/Snail1 pathway-driven EMT was a key signaling event leading to the acquisition of gemcitabine resistance in pancreatic cancer cells [73]. An antiviral drug zidovudine inhibited these signaling pathways and restored the gemcitabine sensitivity in the cancer cells. Co-administration of zidovudine with gemcitabine in mice bearing gemcitabine-resistant pancreatic tumor xenograft significantly suppressed the tumor formation and prevented the cancer cells from acquiring EMT phenotype. Metformin is an old anti-diabetic drug that was accidently found to lower the blood glucose level in the early 1950s. It received a US-FDA approval for the treatment of type-2 diabetes in the mid 1990s. Recently, metformin has received great attention from oncologists as it showed potential anticancer and chemopreventive effects with its action independent on anti-hyperglycemic effects [74,75,76]. Later on, Hirsch et al., reported that metformin selectively targets breast cancer stem cells (BCSCs) [77]. Follow-up studies demonstrated that metformin inhibits CSCs by targeting EMT. Vazquez-Martin and coworkers showed that metformin induced transcriptional reprogramming of BCSCs by decreasing key EMT-TFs, including ZEB1, Twist1 and SNAI2 [78]. In lung adenocarcinoma, metformin was found to inhibit EMT by blocking IL-6/STAT3 axis [79]. Although it is unclear about the direct molecular target of metformin in inhibiting EMT, activation of AMPK may play a role in part in anti-EMT action of this drug [80,81]. Based on its potential anticancer and CSC activities with a favorable safety profile, metformin is heavily investigated in more than 200 human clinical trials for cancer treatment [82]. Table 1 summarizes the recent advances in EMT-targeting small molecule drugs and their target pathways in EMT. In addition to these small molecules developed, a great deal of drug screening effort is ongoing to identify novel EMT inhibitors. Advances in EMT and CSC biology enabled scientists to perform high throughput screening of small molecules using advanced screening platforms. Chua et al. developed an EMT spot migration detection system, which is amenable for high-content screening and used this system to screen growth factor-specific, small molecule EMT inhibitors [83]. Moreover, Aref et al. generated a microfluidic system containing tumor cell spheroids and adjacent endothelial monolayer, which mimics 3D tumor microenvironment. This system was demonstrated to be particularly useful to identify EMT drugs that are effective in complex in vivo tumor microenvironment where different cell types interact [84,85].

Recently, microRNA (miRNA) has been recognized as a target for overcoming EMT-driven drug resistance. miRNAs are a class of small single-strand non-coding RNAs that regulate gene expression by binding to 3′UTR regions of mRNAs [95,96,97]. miRNAs are involved in diverse signaling pathways, including EMT and drug resistance [98,99]. miR-200 family of miRNAs (including miR-200a, miR-200b, miR-200c, miR-141 and miR-429) inhibit EMT by targeting EMT-TFs, ZEB1 and ZEB2 [100,101]. However, ZEB1 and ZEB2 can also inhibit miR-200 family expression, thus forming a negative feedback loop [102,103]. miR-200 family enhanced sensitivity to nintedanib by regulating EMT in NSCLC [104]. miR-223, however, induced EMT in gemcitabine-resistant pancreatic cancer cells by down regulating Fbw7. Inhibition of miR-223 reversed EMT phenotype and significantly enhanced the gemcitabine sensitivity of the cancer cells [105]. Paclitaxel-resistant breast cancer cells displayed EMT phenotype and showed a significant down-regulation of miR-125b. Over-expression of miR-125b reversed EMT phenotype partly by targeting Sema4C, and significantly sensitized the drug resistant breast cancer cells to paclitaxel [106]. More recently, Zhu et al., reported that cisplatin-resistant ovarian cancer cells displayed EMT phenotype with decreased miR-186 expression and increased Twist1 expression. Over-expression of miR-186 in these cells led to a decreased Twist1 expression, reversed EMT phenotype and sensitized the cells to cisplatin treatment [107]. These results demonstrate that miRNAs, by regulating EMT, are attractive candidates for overcoming cancer drug resistance. A number of miRNAs that regulate EMT and cancer drug resistance have been identified and some of them are summarized in Table 2.

## 6. Conclusions and Future Perspectives

The role of EMT in cancer drug resistance has long been suggested. It became clearer when scientists found remarkable similarities in gene expression signatures and marker expression between cells undergoing EMT and CSCs. CSCs are resistant to most of current chemotherapy regimens, making them extremely hard to eradicate [115]. Based on our better understanding of molecular switches and key signaling pathways in EMT, scientists have successfully developed assay systems to analyze EMT phenotype and drug screening. A large effort has been made to identify small molecules and miRNAs that reverse EMT phenotype and, subsequently, drug resistance in cancer. As a result, several small molecules have been discovered as EMT inhibitors that are capable of enhancing chemo-sensitivity of drug-resistant cancer cells. Many of them entered human clinical studies in combination with standard chemotherapies or targeted therapies, including mocetinostat in combination with gemcitabine for metastatic leiomyosarcoma, metformin in combination with cisplatin/radiation for NSCLC, bufalin in combination with gemcitabine for pancreatic cancer, palbociclib in combination with tamoxifen for HR(+)/HER2(−) advanced breast cancer, and disulfiram in combination with gemcitabine for metastatic pancreatic cancer (www.clinicaltrials.gov). Although some of these drugs were proven to be effective in enhancing the efficacy of chemotherapy, toxicity of the EMT inhibitors is still an issue. Small molecule TGFβ inhibitors showed severe cardiotoxicities in animal preclinical models [116,117], allowing only a few small molecule inhibitors, such as galunisertib, to enter clinical investigations [118]. In addition, it is not yet clear about the safety in the long-term use of the EMT inhibitors [119]. This is particularly important if the EMT inhibitors in turn activate MET process that is believed to be involved in cancer metastasis.

EMT-MET has been recognized to play an important role in cancer cell dissemination and distal metastasis. However, the latest studies suggest that despite its role in chemoresistance, EMT may be dispensable for cancer cell metastasis [28,29]. This conclusion, however, is under strong debate as heterogeneity and plasticity of EMT phenotype should be taken into account. Fsp1 may possibly control only a subset of EMT populations [120]. Another two research groups separately reported that at least two distinct EMT phenotypes are involved in cancer metastasis: one with stemness phenotype (Twist1-type) and the other with non-stemness phenotype (Prrx1 type) [47,121]. In the stemness phenotype, an EMT-TF Twist1 is able to dedifferentiate epithelial type cancer cells into non-proliferative, mobile mesenchymal cells (possessing stemness properties). Down-regulation of Twist1, in turn, reversibly redifferentiate the EMT cells into epithelial cell type at the site of metastasis where the cells start colonizing and proliferating [121]. In contrast, in non-stemness EMT phenotype, a newly discovered EMT-TF Prrx1 activates cells to EMT that lacks stemness properties. The down-regulation of Prrx1 is required for MET and to allow gaining stemness traits and colonization of cancer cells at the site of metastasis. Of notes, in both cases, reversible transition of cells between EMT (for dissemination) and MET (for colonization) is critical for cancer metastasis [122]. The heterogeneity of EMT phenotype has also been demonstrated by Biddle and coworkers using a cell surface marker profile of cancer stem cells (CSCs) [45].

The heterogeneity and plasticity of EMT phenotype is not only involved in metastasis, but also in drug resistance. Biddle et al. generated four sub-lines of oral squamous cell carcinoma that have distinct EMT status and plasticity: stable epithelial cells (limited ability to undergo EMT), plastic epithelial cells (enhanced ability to undergo EMT), stable EMT cells (unable to undergo MET) and plastic EMT cells (able to undergo MET) [123]. All epithelial cells were sensitive to paclitaxel, while EMT cells were resistant to paclitaxel, regardless of cell plasticity. However for cisplatin and salinomycin, stable epithelial or EMT cells were sensitive, whilst plastic epithelial or EMT cells were resistant. These data suggested that not only the EMT phenotype is involved in certain drug resistance, but also plasticity of cancer cells across EMT–MET processes is critical for resistance to different classes of drugs. Therefore it would be necessary to delineate EMT inhibitors into subtypes, such as stable EMT inhibitors and plasticity inhibitors. In this view, the plasticity inhibitors may have a great potential in cancer treatment as this type of drugs may prevent both drug resistance and cancer metastasis.

In conclusion, EMT is an important cancer cell phenotype that drives drug resistance. Inhibitors of this cellular process will work as good “partners” for chemotherapy or targeted therapy drugs, which can significantly improve the clinical outcomes of current cancer therapeutics.

## Figures and Tables

**Table 1:** Small molecule inhibitors of epithelial–mesenchymal transition (EMT) and their functions.

Drugs	Target Genes	Function	Cancer	Ref.
Curcumin	BMI1, SUZ12 and EZH2	Inhibits EMT and reverses 5-fluorouracil resistance	Colorectal cancer	[71]
Mocetinostat	HDAC	Induces sensitivity against chemotherapy	Pancreatic cancer	[72]
Zidovudine	Akt-GSK3 beta-Snail pathway	Inhibits EMT and reverses gemcitabine resistance	Pancreatic cancer	[73]
Evodiamine	WNT pathway	Inhibits EMT and reverses oxaliplatin resistance	Gastric cancer	[86]
Pyrvinium pamoate	WNT pathway	Inhibits EMT	Breast cancer	[87]
Moscatilin	Vimentin, Slug, and Snail	Inhibits EMT and sensitizes anoikis	Lung cancer	[88]
Metformin	ZEB1, Slug, Twist and Vimentin	Inhibits EMT	Breast cancer Ovarian cancer	[78,89]
Palbociclib	c-Jun/COX-2	Inhibits EMT	Breast cancer	[90]
Icaritin	PTEN/Akt/HIF-1α pathway	Inhibits EMT	Glioblastoma	[91]
Disulfiram	ERK/NF-kappa B/Snail pathway	Inhibits EMT and stem cell-like features	Breast cancer	[92]
Zerumbone	TGFβ pathway	Inhibits EMT	Non-small cell lung cancer	[93]
Bufalin	TGFβ pathway	Inhibits EMT	Lung cancer	[94]

**Table 2:** miRNAs associated with epithelial–mesenchymal transition (EMT) and their functions.

miRNA	Target Gene	Function	Cancer	Ref.
miR-200	ZEB1, ZEB2	Inhibits EMT and reverses nintedanib resistance	Non-small cell lung cancer	[104]
miR-223	Fbw7	Induces EMT and confers gemcitabine-resistance	Pancreatic cancer	[105]
miR-125b	Sema4C	Inhibits EMT and reverses paclitaxel-resistance	Breast cancer	[106]
miR-186	Twist1	Inhibits EMT and reverses cisplatin-resistance	Ovarian cancer	[107]
miR-15b	PEBP4	Induces EMT and confers cisplatin resistance	Lung adenocarcinoma	[108]
miR-106a	Twist1	Inhibits EMT and reverses gemcitabine resistance	Hepatocellular carcinoma	[109]
miR-203	Slug	Inhibits EMT and reverses imatinib resistance	Glioblastoma	[110]
miR-375	MTDH	Inhibits EMT and reverses tamoxifen resistance	Breast cancer	[111]
miR-27a	RKIP	Induces EMT and confers cisplatin resistance	Lung adenocarcinoma	[112]
miR-489	Smad3	Inhibits EMT and reverses chemoresistance	Breast cancer	[113]
miR-671-5p	FOXM1	Inhibits EMT and reverses cisplatin resistance	Breast cancer	[114]
